# Blood and bone marrow dosimetry for thyroid cancer patients prepared with rhTSH injection

**DOI:** 10.1007/s12149-025-02042-7

**Published:** 2025-04-05

**Authors:** Amna Juma Al Jabri, Jennie Cooke, Seán Cournane, Marie-Louise Healy

**Affiliations:** 1 School of Medicine, Trinity College Dublin, Dublin, Ireland; 2 Department of Radiology and Molecular Imaging, Sultan Qaboos University, Al Koudh, P.O. Box: 25, P.C.: 123 Muscat, Sultanate of Oman; 3 Department of Radiology, Children’S Health Ireland at Crumlin, Dublin, Ireland; 4 Department of Medical Physics and Clinical Engineering, St Vincent’S University Hospital, Dublin, Ireland; 5 Department of Endocrinology, St James’s Hospital, Dublin, Ireland

**Keywords:** Thyroid, Dosimetry, Thyrogen, RhTSH, Cancer, Pretherapy

## Abstract

**Purpose:**

Radioiodine (^131^I) dosimetry is used to maximise tumour dose while reducing the chances of toxicity. High thyroid-stimulating-hormone (TSH) levels are required for ^131^I treatment, achieved through hormone withdrawal or intramuscular injection of recombinant human TSH (rhTSH). Both approaches have shown equivalent results, with the rhTSH approach reported to reduce morbidity and avoid hypothyroidism. There are established differences in ^131^I biokinetics using each method*.* This clinical cohort study investigated if pretherapy iodine biokinetics as measured using a dosimetry protocol without a dose of rhTSH are predictive of post therapy biokinetics in patients prepared with rhTSH injection.

**Methods:**

Thirteen patients with differentiated thyroid cancer (DTC) were recruited. An adaptation of the European Association of Nuclear Medicine (EANM) dosimetry protocol was conducted at St James’s Hospital, Ireland. The maximum tolerable activity (MTA) was calculated using the EANM, Association of Physics in Medicine (AIFM) and Traino models, after administering ^131^I, and subsequent whole-body (WB) dose-rate measurements and blood-sampling were carried out. The MTA estimated from pre-therapeutic (PT) ^131^I tracer administration (6.07 ± 2.46 MBq) was compared to during therapy (DT) administration (3.88 ± 0.16 GBq).

**Results:**

The PT WB residence-time overestimated the DT with a difference of − 7.72 ± 8.13% (*p* = 0.007), while no significant difference is reported between the blood residence-time (1.13 ± 6.49%, *p* = 0.559). The EANM model reported the lowest difference of 1.73 ± 4.83% (*p* = 0.241) in MTA.

**Conclusion:**

This study validated the feasibility of using dosimetry in euthyroid patients to predict therapeutic ^131^I biokinetics in DTC patients prepared with rhTSH.

## Introduction

Since the 1940s, radioiodine (^131^I) has been used for the ablation of thyroid cells remaining after thyroidectomy and in the case of disease recurrence. ^131^I therapy was originally introduced with a personalised approach, however, the use of fixed activities has become common practice [1]. Personalised ^131^I therapy for remnant ablation post-surgery is now directed by the European Union (EU) Council directive 2013/59/Euratom [2]. Recent studies have shown how a personalised dosimetric approach can lead to improved outcomes in metastatic differentiated thyroid cancer (MTC), with the feasibility and importance of the dosimetry protocol having been validated [3, 4].

The European Association of Nuclear Medicine (EANM) dosimetry committee published a standard operational procedure (SOP) based on the Benua–Leeper approach [5], providing guidelines for personalising iodine (^131^I) treatment through the pretherapeutic assessment of radioiodine biokinetics of thyroid remnants post thyroidectomy [6]. The SOP, which implements a blood and bone marrow dosimetry protocol, aims to optimise the therapeutic dose with the bone-marrow absorbed dose being the limiting factor [6], since the bone-marrow is regarded as the most radiosensitive organ in the absence of lung metastases [5, 7]. The effective radiation dose to the bone marrow is estimated by measuring the blood activity level, since the blood is regarded as a surrogate to red bone marrow (RM) [8]. The rationale of the EANM SOP is to maximize absorbed doses to iodine-avid cells without causing toxicity to the bone marrow, in the absence of lung metastases [6]. Administering the maximum therapeutic activity or, rather, maximum tolerable activity (MTA) increases the chance of survival in high risk patients using as little fractionation as possible [6, 9, 10].

In DTC, it is recommended that patients be withdrawn from thyroid hormones before ^131^I administration for remnant ablation, or for subsequent monitoring post therapy, to stimulate thyroid-stimulating hormone (TSH) production [11]. An alternative approach for exogenous TSH elevation is achieved using recombinant human thyrotropin (Thyrogen™ or rhTSH), injected 24 and 48 h before ^131^I therapy administration. This approach is particularly effective for patients who are unable to tolerate hormone withdrawal, or whose hormone withdrawal plan does not lead to TSH elevation.

The ^131^I biokinetics in rhTSH-prepared patients have been compared to those of hormone withdrawal patients in several studies [12–14]. The rhTSH approach has the advantage of improving the quality of a patient’s life by avoiding hypothyroid symptoms caused by hormone withdrawal. Further, the rhTSH approach is reported to lead to a longer remnant ^131^I half-life with faster clearance in the remaining organs and, hence, a reduction in radiation exposure to healthy organs [12]. Faster clearance would lead to a shorter hospital stay which has a positive impact in the clinical setting and on economic cost. The rhTSH radioiodine therapy approach has been evidenced to lead to a 57% reduction in hospital costs due to reduced hospitalisation durations [15]. Lower blood absorbed doses have also been reported in patients administered with rhTSH, relative to hormone withdrawal patients [13, 14], facilitating an increase in the administered activity without inducing bone-marrow toxicity.

In benign thyroid disease, pre-therapy (PT) dosimetry has been proven to be predictive of during therapeutic (DT) ^131^I biokinetics, if a reliable thyroid gland estimate is performed and the kinetics before and during therapy are matched [16]. In DTC, PT dosimetry has been reported to be predictive of DT dosimetry for patients in a hormone withdrawal regime [17], but there are limited data on outcomes for patients treated using the rhTSH approach. A percentage difference of − 1.0% ± 18.0% (*p* = 0.401) between PT and DT dosimetry has been reported for 62 metastatic DTC treatments after 4 weeks of hormone withdrawal, with the EANM approach used [17]. The comparison also employed other approaches, such as the Association of Physics in Medicine (AIFM) and Traino models, with a − 2.5% ± 16.4% difference reported for both. There are limited studies comparing PT and DT approaches where patients have been prepared with rhTSH, with low number of patients typically involved. In a study by Verburg et al. [9], only one patient out of 13 treatments was prepared with rhTSH, with the study reporting a ± 25% difference between PT and DT in 92% of the cases. Another study, by Bianchi et al. [18], investigated the impact of patient preparation on the MTA. For this, 27 metastatic DTC patient treatments, of which 11 were prepared using rhTSH, were examined and it was found that the percentage difference between PT and DT dosimetry was − 10% to 284%. The authors attributed the reported difference to their measurement device and to measurement protocol limitations. A larger sample of patients prepared with rhTSH is needed to validate the predictability of the PT dosimetry to facilitate personalised treatment using the Benua–Leeper approach, irrespective of the patient preparation method.

Accordingly, in this study an adapted version of the blood and bone marrow EANM SOP [6] for DTC was investigated by calculating the predictive power of pre-therapy dosimetry in patients prepared by rhTSH injections, to facilitate the implementation of a personalised dosimetry programme using maximum tolerable activities (MTAs) for patients under a euthyroid regime.

## Material and methods

The study was approved by the Tallaght University Hospital/ St James’s Hospital Joint Research Ethical committee (REC: 2019-08 List 31 (04)). The study included patients diagnosed with DTC or MTC and referred to the Nuclear Medicine department at St James’s Hospital, Ireland, for ^131^I ablation post thyroidectomy. An adaptation of the EANM dosimetry protocol [6] was implemented at two stages: PT and DT dosimetry. The patients underwent the same regular treatment plan using a fixed activity approach. 17 patients were recruited for this study, between November 2019 and November 2021. Of the 17 patients, 13 patients had complete pre-therapy and during therapy data sets and contributed to the analysis of this study.

The patient demographical data were also collected. The collected parameters and results of the study included age, gender, whether the patient had MTC or not, whether the treatment was their first therapeutic treatment or a repeat ^131^I therapy, body mass index, PT, DT administrated activities, whole-body and blood residence time in hours, EANM [6], AIFM [19] and Traino [20] maximum tolerable activities in GBq.

The EANM SOP for thyroid cancer therapy requires the measurement of two compartments, the blood (BL) and whole-body (WB) activities. Thus, the ^131^I biokinetics were monitored at a series of time points according to the timelines presented in Table 1. The timing was chosen to accommodate the workflow of the department, it was not expected to have any impact on the result since the only deviation was in the last two time-points, ensuring that the uncorrected WB retention in the last point did not exceed 5% [6].

**Table 1:** Time line of measurements as implemented in St James’s hospital, Ireland, with slight variation from EANM SOP to accommodate the department workflow

Time (h)	Measurement
0	Administration of ^131^I
2	Whole-body activity measurement, blood sampling (2 mL), having avoided micturition or defecation
6	Micturition, whole-body activity measurement, blood sampling (2 mL)
24	Micturition, whole-body activity measurement, blood sampling (2 mL)
48	Micturition, whole-body activity measurement, blood sampling (2 mL)
120	Micturition, whole-body activity measurement, blood sampling (2 mL)
> 120	If uncorrected whole-body retention at 120 h exceeds 5%, then: micturition, whole-body activity measurement, blood sampling (2 mL)

For WB measurements, a geometrical mean was collected for conjugate views, and all the measurements were normalised to the 2 h WB measurements, regarded as 100% of the administered activity, to estimate the activity retention in the WB as a function of time. For blood sampling, 2 mL samples were withdrawn at each time-point with 1 mL extracted for radioactivity quantification, where each sample was normalised to the administered activity to estimate the activity retention per millilitre of blood.

### Pretherapy (PT) dosimetry

For PT dosimetry, patients were administered 2–10 MBq ^131^I-NaI, with their blood and WB data collected according to the timeline in Table 1. The WB probe measurements were taken using a partially shielded thyroid uptake probe (Biodex Atomlab*™*). Venous blood samples were collected in LH Lithium Heparin vials (VACUETTE^®^, 3.5 mL). The net count rate in counts per minute (cpm) and 95%-error were calculated using Eqs. (1) and (2) below.

1 $$\text{Net count rate }\left(\text{cpm}\right)=\left[\text{volume correction}\right]\times \left[B\text{ kg correction}\right]\times \left[\text{decay correction}\right]= \left[\frac{\text{blood volume }(\text{mL})}{\text{measured blood volme }(\text{mL})}\right]\times \left[\frac{\text{gross counts}-\text{background counts}}{\text{counting time }(\text{minutes})}\right]\times \left[{e}^{time(minutes)\times \text{ln}(2)/{T}_{1/2}}\right]$$

2 $$95\% \text{ Error } \left(\%\right)=1.96\times 100\times \frac{\sqrt{\text{gross count}}}{\text{gross count}}$$

where the time is the difference between the counting time and the blood withdrawal, and *T*_1/2_ is the ^131^I half-life of 8.02 days.

The activity retention per mL of blood was deduced according to the EANM SOP [6].

For each blood sample, corresponding WB probe measurements were acquired at the same time point. The thyroid uptake probe was used for pre-therapy measurements. As the probe is partially shielded, patients were positioned at 2 m [6].

### During therapy (DT) dosimetry

For DT dosimetry, patients were administered 3.7 GBq ^131^I-NaI. The blood samples were collected as per the PT procedure, according to the timeline in Table 1. The WB measurements were performed more frequently due to the continuous availability of the patient in the room and the potential to increase the accuracy of the fitting. All WB measurements were taken by the patients themselves using an a NaI count rate meter (Gamma Probe, FHZ 621 G-L4-10, Thermo Fisher Scientific Messtechnik GmbH) fixed to the wall of the patient isolation suite.

The WB measurements were relative measurements, normalised to the first acquired data point. Different measurement devices were used for the PT and DT WB measurements. However, this was not expected to cause variation in the PT vs DT comparison given that the same probe was used for the PT and DT measurements [6, 21].

The activity retention for both WB and BL was used to calculate the residence time (*τ*) within the WB (*τ*_WB_) and blood (*τ*_BL_) according to the EANM SOP [6]. The MIRD formalism [22], which is the basis of personalising ^131^I treatment as published by EANM [6], states that the blood absorbed dose per administered activity (${{\overline{D}} \mathord{\left/ {\vphantom {{\overline{D}} {A_{0} }}} \right. \kern-0pt} {A_{0} }}$) is composed of a contribution from blood self-irradiation and WB radiation (Eq. 3):

3 $$\frac{\overline{D}}{{A }_{0}}={S}_{\text{BL}\leftarrow \text{BL}}\cdot {\tau }_{\text{BL}}+S_{\text{BL}\leftarrow \gamma \text{WB}}\cdot {\tau }_{\text{WB}}$$

where *S*_BL←BL_ is the blood contribution and *S*_BL_ _←_ *γ*_WB_ is the *γ* contribution from the WB to the *τ* [23]. The ${{\overline{D}} \mathord{\left/ {\vphantom {{\overline{D}} {A_{0} }}} \right. \kern-0pt} {A_{0} }}$ in this study was calculated according to Eq. (4).

4 $$\frac{{\overline{D}_{{{\text{BL}}}} }}{{A_{0} }}\left[ {\frac{{{\text{Gy}}}}{{{\text{GBq}}}}} \right] = 108 \cdot \tau_{{{\text{BL}}}} \;\left[ {\text{h}} \right] + \frac{0.0188}{{\left( {{\text{wt}}\;\left[ {{\text{kg}}} \right]} \right)^{\frac{2}{3}} }} \cdot \tau_{{{\text{WB}}}} \;\left[ {\text{h}} \right]$$

The EANM SOP recommends using Eq. (4) for calculating the MTA. However, this is under the conservative assumption that the dose to the blood is identical to the haematopoietic tissues [8]. The AIFM and Traino models for RM absorbed dose [19, 20] were also used for calculating RM absorbed doses per administered activity. Equations (5) and (6) were used to calculate the RM absorbed doses per activity using the AIFM models [19] for female and male patients, respectively.

5 $$\frac{{\overline{D}_{{{\text{RM}}}} }}{{A_{0} }}\left[ {\frac{{{\text{Gy}}}}{{{\text{GBq}}}}} \right] = 65 \cdot \tau_{{{\text{BL}}}} \;\left[ {\text{h}} \right] + \frac{0.0945}{{\left( {{\text{wt}}\;\left[ {{\text{kg}}} \right]} \right)}} \cdot \tau_{{{\text{WB}}}} \;\left[ {\text{h}} \right]$$

6 $$\frac{{\overline{D}_{{{\text{RM}}}} }}{{A_{0} }}\left[ {\frac{{{\text{Gy}}}}{{{\text{GBq}}}}} \right] = 61 \cdot \tau_{{{\text{BL}}}} \;\left[ {\text{h}} \right] + \frac{0.105}{{\left( {{\text{wt}}\;\left[ {{\text{kg}}} \right]} \right)}} \cdot \tau_{{{\text{WB}}}} \;\left[ {\text{h}} \right]$$

Equations (7) and (8) were used to calculate the RM absorbed doses per activity using the Traino models [20] for female and male patients, respectively.

7 $$\frac{{\overline{D}_{{{\text{RM}}}} }}{{A_{0} }}\left[ {\frac{{{\text{Gy}}}}{{{\text{GBq}}}}} \right] = 58.97 \cdot \tau_{{{\text{BL}}}} \;\left[ {\text{h}} \right] \cdot {\text{wt}}\;\left[ {{\text{kg}}} \right]^{0.028} + \left( {\tau_{{{\text{WB}}}} \;\left[ {\text{h}} \right] - 22.8 \cdot \tau_{{{\text{BL}}}} \;\left[ {\text{h}} \right] \cdot {\text{wt}}\;\left[ {{\text{kg}}} \right]} \right) \cdot \left[ {\frac{0.5427}{{{\text{wt}}\;\left[ {{\text{kg}}} \right]^{1.302} }} - \frac{3.4074}{{{\text{wt}}\;\left[ {{\text{kg}}} \right]^{1.944} }}} \right]$$

8 $$\frac{{\overline{D}_{{{\text{RM}}}} }}{{A_{0} }}\left[ {\frac{{{\text{Gy}}}}{{{\text{GBq}}}}} \right] = 55.89 \cdot \tau_{{{\text{BL}}}} \;\left[ {\text{h}} \right] \cdot {\text{wt}}\;\left[ {{\text{kg}}} \right]^{0.026} + \left( {\tau_{{{\text{WB}}}} \;\left[ {\text{h}} \right] - 15.2 \cdot \tau_{{{\text{BL}}}} \;\left[ {\text{h}} \right] \cdot {\text{wt}}\;\left[ {{\text{kg}}} \right]} \right) \cdot \left[ {\frac{0.6967}{{{\text{wt}}\;\left[ {{\text{kg}}} \right]^{1.331} }} - \frac{4.1683}{{{\text{wt}}\;\left[ {{\text{kg}}} \right]^{1.948} }}} \right]$$

Using $\frac{{\overline{D} }_{\text{BL}}}{{A}_{0}}$ and $\frac{{\overline{D} }_{\text{RM}}}{{A}_{0}}$, the MTA can be calculated using Eq. (9) below.

9 $$\text{MTA }\left[\text{GBq}\right]=\frac{2}{{\overline{D} }_{\text{BL}}/{A}_{0}\;[\text{Gy}/\text{GBq}]}$$

Both blood and RM absorbed dose values were reported in this study, corresponding to the EANM (blood) [6], AIFM [19] and Traino [20] (RM) approaches, respectively.

### Pre-therapy vs during therapy dosimetry comparison

The activity retention per time data were used to extract fitting constants to calculate residence time (*τ*) and MTA according to the EANM SOP [6] and Eqs. (1) to (9).

The differences investigated in this study were:

- The percentage (%) difference in *τ*_WB_ between PT and DT (referred to as ∆*τ*_WB_)
- The % difference in *τ*_BLOOD_ between PT and DT (referred to as ∆*τ*_BLOOD_)
- The % difference between the blood-based PT-MTA and DT-MTA (referred to as ∆MTA_EANM_)
- The difference between the RM-based PT-MTA and DT-MTA (referred to as ∆MTA_AIFM_ and ∆MTA_Traino_).

The measurement uncertainty was calculated using a propagation of errors approach [24], where this scheme was detailed in the EANM practical guidelines on uncertainty analysis on MRT [25]. The uncertainty in activity retention was estimated by minimising the weighted sum of squares residual (SSE) [26]. For this, a weighting proportional to 1/∆*R*(*t*)^2^ was applied to each time point, such that the weighted SSE = (*R*(*t*)_observed_ − *R*(*t*)_predicted_)^2^/∆*R*(*t*)^2^), where *R*(*t*)_observed_ was the measured fraction of estimated activity and *R*(*t*)_predicted_ was the calculated fraction based on fitting parameters.

### Statistical analysis

The statistical analysis was completed using the web-based Minitab®2021. An Anderson–Darling normality test was used to test the normality assumption of the differences ∆*τ*_WB_, ∆*τ*_BLOOD_, ∆MTA_EANM_, ∆MTA_AIFM_ and ∆MTA_Traino_, where the null hypothesis was that the percentage differences were normally distributed. A Grubb’s test was also applied to these percentage differences to detect for possible outliers in the data, where the null hypothesis assumed all data values came from the same normal population. Any detected outliers were discussed and removed. A paired sample *t*-test was used to compare the PT vs DT variables. Additionally, linear regression analysis was performed to estimate the DT residence times (*τ*_WB_ and *τ*_BLOOD_) using the PT dosimetry results. Bland–Altman analysis was conducted on the PT and DT MTAs. The analysis plots included the difference between PT and DT results, compared to their average, the mean differences and their 95% confidence interval (95% limit of agreements). A significance level (*p*-value) of 0.05 was used in all the analysis.

## Results

The study sample included 13 patients: 5 males and 8 females, 5 patients with metastatic disease (38%) and 4 with repeated treatment (31%). Table 2 shows normality and outlier test results of the differences of the investigated parameters with ∆*τ*_BLOOD_, ∆MTA_EANM_, ∆MTA_AIFM_ and ∆MTA_Traino_ rejecting the normality assumption (*p* < 0.05).

**Table 2:** Normality test as performed in percentage difference between pretherapy vs during therapy for the parameters: whole-body and blood residence times (∆*τ*_WB_ and ∆*τ*_BLOOD_), EANM, AIFM and Traino maximum tolerable activities (∆MTA_EANM_, ∆MTA_AIFM_ and ∆MTA_Traino_)

Anderson–Darling normality test	
Parameter (%)	*p*-value
∆*τ*_WB_	0.334
∆*τ*_BLOOD_	0.008
∆MTA_EANM_	< 0.005
∆MTA_AIFM_	0.028
∆MTA_Traino_	0.028

Grubb’s test or maximum normalised residual test		
Patient no	Outlier identified	*p*-value
P7	∆*τ*_BLOOD= −40.61_	0.001
P7	∆MTA_EANM=36.28_	< 0.001
P7	∆MTA_AIFM=32.84_	0.003
P7	∆MTA_Traino=32.74_	0.003

Anderson–Darling normality test after omitting outlier	
Parameter (%)	*p*-value
∆*τ*_WB_	0.458
∆*τ*_BLOOD_	0.782
∆MTA_EANM_	0.837
∆MTA_AIFM_	0.788
∆MTA_Traino_	0.717

**Fig. 1:**
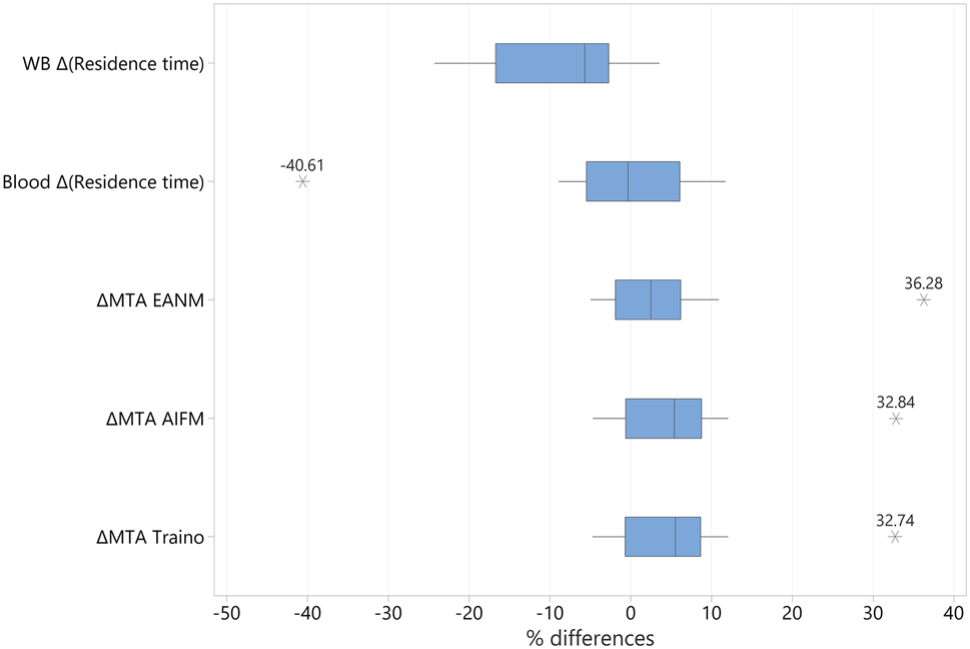
Box plot of the percentage differences (∆) between the pre-therapy and during therapy parameters: whole-body (WB) and blood residence times, EANM, AIFM and Traino maximum tolerable activities (MTA). Each box shows the interquartile range box, range lines and the outliers displayed with numbers

Figure 1 displays the interquartile range and identified outliers, with the ∆*τ*_WB_ showing a wider spread than the blood results, and the EANM approach having a narrower range in ∆MTA as compared to AIFM and Traino approaches.

In testing for outliers in these parameters, P7 (referred to as patient number 7 in order of patient recruitment) was identified as being an outlier and was omitted from the analysed sample. P7 was a 66 years old male with stage IV chronic kidney disease, in addition to metastatic thyroid cancer, secondary ANCA (anti-neutrophilic cytoplasmic autoantibodies) vasculitis and pre-existing glomerulonephritis. It was anticipated that his results would deviate from the rest of the studied sample, given his diagnosis, and this was confirmed by the outlier test. His eGFR fluctuated between 22 and 28 mL/min/1.73 m^2^ in PT to DT period, with creatine reported as 210–257 μmol/L and urea as 11.6–15.5 mmol/L (reference ranges for adult male is 53–106 μmol/L for creatinine and 1.8–7.1 mmol/L for urea [27]). The patient eGFR changed between the PT and DT period due to his compliance with a special renal diet as advised by the renal medicine consultant, which led to a large difference in PT values when compared to DT, $\Delta {\text{MTA}}_{\text{EANM}}$ was –36.8% ($\Delta {\text{MTA}}_{\text{AIFM}}$ and $\Delta {\text{MTA}}_{\text{Traino}}$ was ≈ 32%). There were no other significant patient outliers identified. On repeat of the normality test, the normality assumption was considered valid and the remaining sample was used for subsequent analysis.

Summary statistics are reported for the 12 patients sample in Table 3. For each parameter, the average, standard deviation, range (minimum and maximum) and *p*-value for paired sample *t*-tests were calculated.

**Table 3:** Summary of the collected parameters for a sample of 12 patients, 4 males and 8 females

Parameter	Mean ± StDev	Minimum	Maximum	*p*-value
Patient data				
Age (years)	50.08 ± 16.43	30.30	72.79	
Male	61.27 ± 10.81	45.30	69.05	
Female	44.48 ± 16.34	30.30	72.79	
BMI (kg/m^2^)	27.44 ± 6.24	15.44	40.43	
Male	32.41 ± 5.65	28.09	40.43	
Female	24.96 ± 5.14	15.44	32.74	
PT activity (MBq)	6.07 ± 2.46	2.00	9.02	
DT activity (GBq)	3.88 ± 0.16	3.67	4.11	
Dosimetry results				
PT *τ*_WB_ (h)	20.15 ± 7.95	12.63	37.46	0.027
DT *τ*_WB_ (h)	18.29 ± 5.77	11.92	29.35	
PT *τ*_BLOOD_ × 10^–4^ (h)	5.75 ± 1.90	3.62	9.92	0.798
DT *τ*_BLOOD_ × 10^–4^ (h)	5.77 ± 1.77	3.75	9.69	
PT MTA_EANM_ (GBq)	25.47 ± 6.37	14.55	38.25	0.346
DT MTA_EANM_ (GBq)	25.84 ± 6.01	14.91	37.54	
PT MTA_AIFM_ (GBq)	33.48 ± 8.98	19.44	54.11	0.081
DT MTA_AIFM_ (GBq)	34.47 ± 8.36	19.96	53.65	
PT MTA_Traino_ (GBq)	34.26 ± 8.96	20.19	53.68	0.086
DT MTA_Traino_ (GBq)	35.25 ± 8.29	20.72	53.22	
PT ${\overline{D} }_{\text{BLOOD}}/{A}_{0}$ (Gy/GBq)	0.09 ± 0.03	0.05	0.15	0.945
DT ${\overline{D} }_{\text{BLOOD}}/{A}_{0}$ (Gy/GBq)	0.09 ± 0.03	0.06	0.14	
PT ${\overline{D} }_{\text{RM}}/{A}_{0}$ (Gy/GBq)^a^	0.07 ± 0.02	0.04	0.11	0.395
DT ${\overline{D} }_{\text{RM}}/{A}_{0}$(Gy/GBq)	0.06 ± 0.02	0.04	0.10	
DT ${\overline{D} }_{\text{BLOOD}}$ (Gy)	0.33 ± 0.07	0.24	0.51	0.011^b^
DT ${\overline{D} }_{\text{RM}}$ (Gy)^a^	0.25 ± 0.06	0.17	0.38	
Percentage difference between PT and DT dosimetry				
∆*τ*_WB_ (%)	− 7.72 ± 8.13	− 24.28	3.53	0.039^c^
∆*τ*_Blood_ (%)	1.13 ± 6.49	− 8.94	11.72	0.559
∆MTA_EANM_ (%)	1.73 ± 4.83	− 4.99	10.90	0.241
∆MTA_AIFM_ (%)	3.35 ± 5.29	− 4.71	12.03	0.051
∆MTA_Traino_ (%)	3.31 ± 5.33	− 4.73	12.05	0.055

Displayed mean ± standard deviation, minimum, maximum and a *p*-value for pair *t*-test between pretherapy (PT) vs during therapy (DT)

^a^The red bone marrow absorbed doses (RM) is based on the AIFM approach

^b^This *p*-value corresponds to two sample *t*-test between DT ${\overline{D} }_{\text{BLOOD}}$ and DT ${\overline{D} }_{\text{RM}}$

^c^A non-parametric 1-sample sign test was used since the normality of differences was not assumed

In comparing the dosimetry results, only *τ*_WB_ showed a significant difference between PT and DT (*p* = 0.027). This may have been due to the difference in the number of PT and DT WB measurements acquired, with 5 WB measurements taken in the PT dosimetry period and 15 measurements acquired in the DT dosimetry period. Thus, the uptake curves contained a more highly sampled dataset. The *τ*_BLOOD_ values were not significantly different between the PT and DT procedures. The percentage differences between the PT vs DT values were again significantly different from 0 for ∆*τ*_WB_ (*p* = 0.039), while they were not significantly different for ∆*τ*_BLOOD_, ∆MTA_EANM_, ∆MTA_AIFM_ and ∆MTA_Traino_.

The EANM and AIFM models were used for comparing the absorbed dose using the blood based model versus a RM based model as both the AIFM and Traino models reported results that were not significantly different from each other. A comparison of MTA_AIFM_ vs MTA_Traino_ using a two-sample *t*-test reported *p* = 0.834 for PT and *p* = 0.821 for DT. The MTA_*EANM*_ values were consistently lower than those of the MTA_AIFM_ model. The DT ${\overline{D} }_{\text{BLOOD}}$ values were higher than those of DT ${\overline{D} }_{\text{RM}}$ (*p* = 0.011, two sample *t*-test). There was no reported potential toxicity in this cohort with a maximum blood absorbed dose of 0.51 Gy and a RM absorbed dose of 0.38 Gy estimated.

**Fig. 2:**
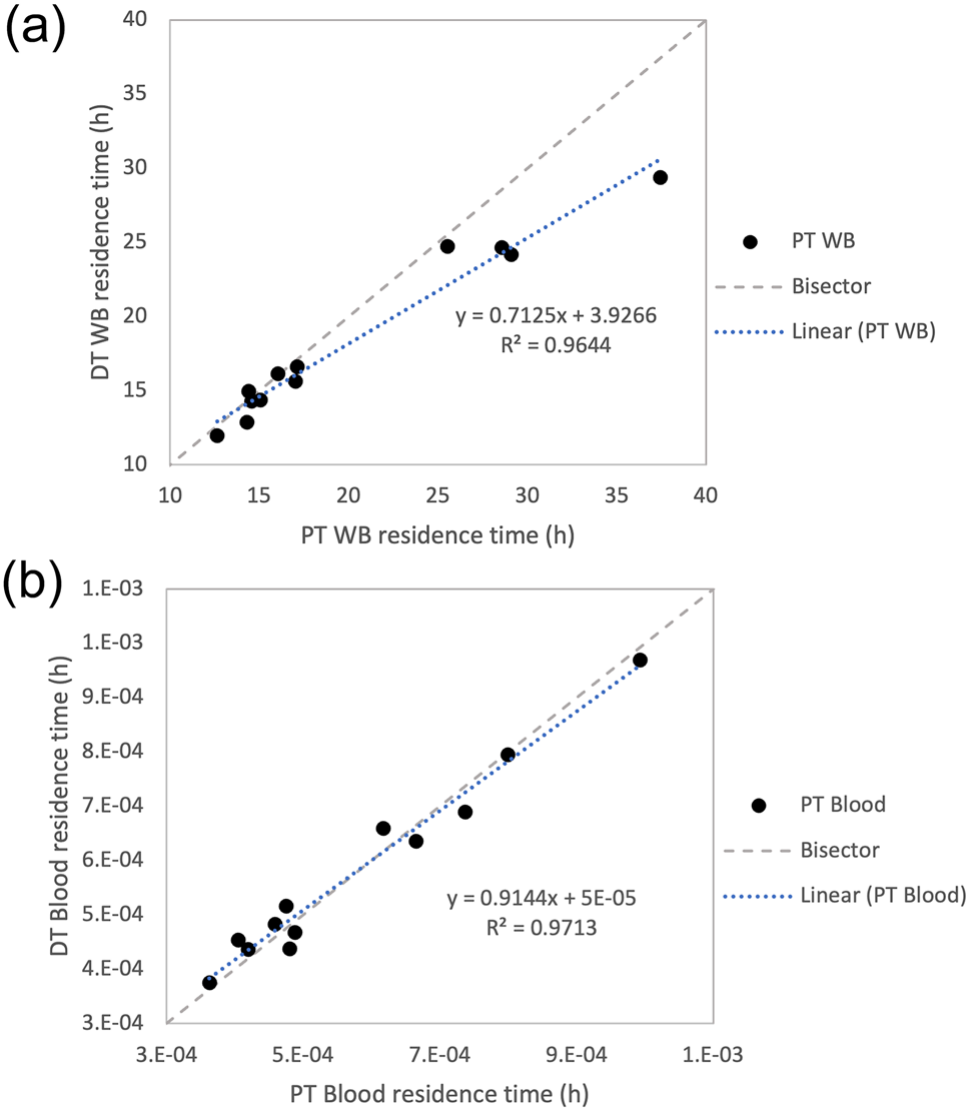
Scatter plot of the therapeutic (DT) residence time and pretherapy results (PT) for both WB (**a**) and blood (**b**). Also displayed is a linear fit for the point, regression model with coefficient of determination (*R*^2^) and bisector of the graph

A linear regression model was applied for the PT and DT *τ*, to examine the accuracy of estimating the therapeutic results based on PT dosimetry. Table 4 shows high values of *R*^2^ and $R_{{{\text{pred}}}}^{2}$ which indicates that a high percentage of data points can be explained by the linear model. 96.44% of DT *τ*_WB_ can be explained by PT *τ*_WB_ with 94.01% predictability. The blood reported higher predictability of 96.21% between DT and PT residence time. Also, Fig. 2 displays a scatter plot of DT versus PT residence time. The points were seen to be close to the bisector line. For the WB measurements, most points fell below the bisector line. This was not seen in the blood residence time plot.

**Table 4:** Linear regression model for the therapeutic residence time (DT *τ*) and pretherapy residence time (PT *τ*) for WB and blood, with *p*-value < 0.001 for the slope but non-significant constant (*p* = 0.861 for WB and 0.578 for blood)

Regression model	*N*	*R*	*R*^2^	$R_{{{\text{pred}}}}^{{2}}$
$\text{DT } {\tau }_{\text{WB}}=3.927+0.7125 \text{ PT } {\tau }_{\text{WB}}$	12	0.982	96.44%	94.01%
$\text{DT } {\tau }_{\text{BLOOD}}=5.2 \times {10}^{-5}+0.9144 \text{ PT } {\tau }_{B\text{LOOD}}$	12	0982	97.13%	96.21%

The table display the sample size (*N*), correlation coefficient (*r*), the coefficient of determination (*R*^2^) and its predictive value ($R_{{{\text{pred}}}}^{{2}}$)

**Fig. 3:**
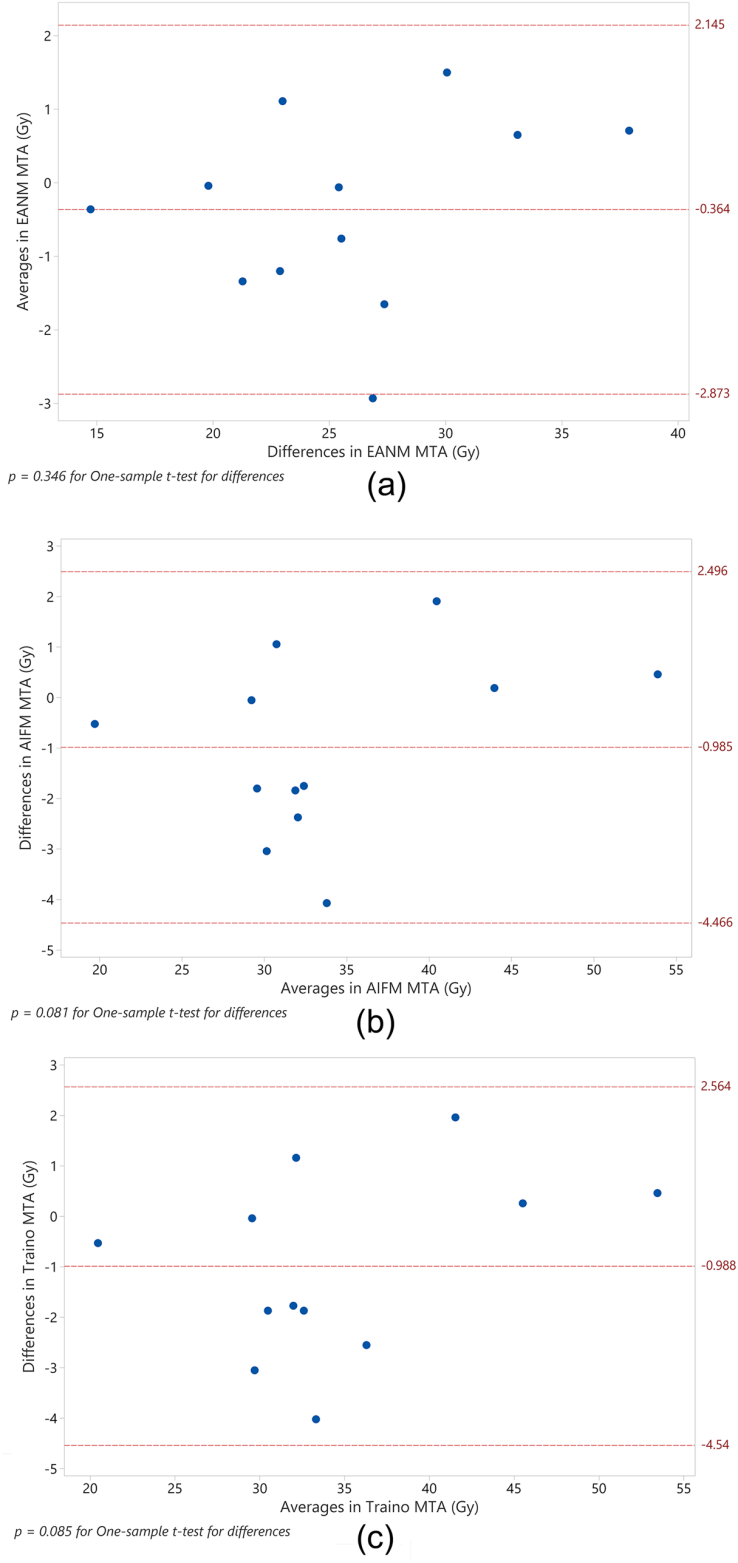
Bland–Altman plots for differences versus averages in activity for EANM (**a**), AIFM (**b**) and Traino (**c**) maximum tolerable activities (MTA). Also displayed are three reference lines representing the mean, ± 95% limits of agreement

Finally, Bland–Altman analysis was used to examine the differences between PT and DT results in ∆MTA(*GBq*), as compared to the averages. Figure 3 shows Bland–Altman plots of the differences versus averages of the plotted parameters, displaying the mean of differences and the ± 95% limit of agreement. Figure 3 also displays *p*-values for the one-sample *t*-test for the differences which were all shown to have non-significant differences from zero. The plots show a spread of most data points within the 95% limit of agreement, which fall within ± 5% of the differences.

## Discussion

The two dosimetry approaches for DTC using ^131^I are bone-marrow dose limiting dosimetry and lesion-based dosimetry. The first is the main focus of this study and considers the safety of the administered activity while limiting the absorbed dose to the critical organ, which is the bone-marrow in the absence of lung metastasis. Both approaches rely on PT dosimetry to calculate patient-specific treatments, and it has been shown that the ^131^I biokinetics in PT and DT are analogous for particular cohorts and treatment approaches [17, 28]. This assumption, however, is in need of validation in the case of patients prepared for ^131^I therapy using rhTSH. In this work, the feasibility of using dosimetry in euthyroid patients to predict therapeutic ^131^I biokinetics in DTC patients prepared with rhTSH is demonstrated.

The collected dosimetry data were used to estimate WB and blood residence times (*τ*_WB_ and *τ*_BLOOD_, respectively) for both PT and DT stages, with the percentage difference between the two then calculated. The result reported high values of percentage difference between PT *τ*_WB_ relative to DT, while for the blood there was no significant difference between PT and DT data. The blood measurements in both PT and DT were measured using the same well counter, considering dead-time values and statistical variation across different days. Additionally, similar sampling regimes were carried out. For the WB in DT, there was a mismatch in the sampling frequency and, in addition, measurements were performed on different devices which may have led to further differences. Furthermore, patient self-measurement of WB DT might be a contributing factor to this difference.

Despite the significant differences in *τ*_WB_, there was no significant difference detected in the MTA for the models. The EANM model had a lower % difference between PT vs DT, while the AIFM and Traino models returned *p*-values close to the set significant level of 0.05 (*p* = 0.051 and 0.055, respectively). This is likely caused by the differing weighting of the blood absorbed dose contribution in each model. The EANM model considers a 75% contribution from the blood absorbed dose while it is 55% in the RM models [17]. In all of the patients, the MTA_AIFM_ and MTA_Traino_ values were higher than the MTA_EANM_. This would be expected, since the EANM model is regarded as a more conservative approach. The blood absorbed dose after therapeutic administration of ^131^I was statistically higher in the EANM model than the RM absorbed doses of the AIFM and Traino models. The average blood absorbed doses were 0.33 ± 0.07 Gy for the blood based approach and 0.25 ± 0.06 Gy for the RM approach (*p* = 0.011, two sample *t*-test), a difference which is in keeping with the literature [6, 29].

Fluctuation in kidney function may result in a large difference in ^131^I retention [30, 31] and, in one patient in this study, led to the PT being poorly correlated with DT results. Patients with similar diagnosis require safe limit assessments before administering therapeutic ^131^I and, thus, dosimetry should be applied in parallel to continuous monitoring of eGFR and creatinine level, as changes in function may affect the accuracy of the estimated results. Three patients from our sample were aged > 66 years with MTC and having had multiple unsuccessful ^131^I treatments. A prior ^131^I treatment has been linked to a change in iodine biokinetics leading to remnants becoming more radio-resistant [32]. It is suggested that this can be prevented by administering higher MTA [33] with dosimetry approaches allowing for the estimation of safe limits to critical organs. In previous studies, calculated MTA values have been reported to be safe and tolerable in DTC and MTC patients [34].

Giostra et al. [17] compared PT vs DT dosimetry results for 50 MTC patients using the three dosimetric approaches employed in our study, except their patient cohort was prepared with 4 weeks of hormone withdrawal. Their results showed PT *τ*_WB_ overestimated the residence time compared to DT, which is similar to our study. In addition, all reported values including residence times, absorbed doses per administered activity and MTAs were not significantly different from our data (*p* > 0.05, two-sample *t*-test), except with the DT *τ*_WB_, where our study values were 19% lower (*p* = 0.043, two-sample *t*-test). It would be expected that hormone withdrawal patients would exhibit reduced ^131^I clearance times given that hypothyroidism affects renal function. Indeed, effective half-life values for rhTSH patients have been shown to be reduced by 31% as compared with those not prepared with rhTSH [35]. The Giostra study reported a much larger range of parameters than presented here, with differences of up to − 48% reported in ∆*τ*_BLOOD_. The maximum difference in ∆*τ*_BLOOD_ observed in this current study was − 24.28% while the maximum ∆MTA was 12.05% [17].

The predictive power of PT can be largely affected by the instruments used. A reported high percentage difference between PT and DT due to differences in measurement setup has previously been reported [28]. This cause of outliers was minimised in this current study by replicating measurements setup in both PT and DT.

One of the study's limitations is the small patient sample size. The PT dosimetry commitment required by patients is one of the main factors contributing to the patients being unable to join the study, as it requires repeated hospital visits over 3 days and lengthy stays. Nevertheless, for this small cohort, the statistical analyses are robust and demonstrate significance. In addition, coexisting medical conditions can affect data collection and, hence, the predictive power of PT in patients with known co-morbidities impacting their clearance. Slower renal clearance would require sampling after 120 h, with a longer gap between PT and DT needed.

In conclusion, the results of this study show a strong predictability of the PT dosimetry in absorbed dose calculation for DTC and MTC using rhTSH preparation, where acceptable differences were found in PT vs DT results. Implementing high activities has been demonstrated to be safe by several centres [28], but more caution should be exercised for patients with co-existing illnesses. The protocol implemented in this study shows the potential to perform dosimetry with minimum staff involvement in data collection, since WB measurements were conducted easily by the patient.

## Data Availability

The datasets generated during and/or analysed during the current study are available from the corresponding author on reasonable request.
